# (2*E*)-2-Benzyl­idene-9-phenyl-3,4-di­hydro­acridin-1(2*H*)-one

**DOI:** 10.1107/S1600536814015943

**Published:** 2014-07-17

**Authors:** T. Vinuchakkaravarthy, M. Sankaran, P. S. Mohan, D. Velmurugan

**Affiliations:** a Centre of Advanced Study in Crystallography and Biophysics, University of Madras, Maraimalai (Guindy) Campus, Chennai 600 025, India; b Department of Chemistry, Bharathiar University, Coimbatore 641 046, India

**Keywords:** crystal structure

## Abstract

In the title compound, C_26_H_19_NO, the plane of the aromatic heterocycle makes a dihedral angle of 75.22 (4)° with that of the attached phenyl ring. In the crystal, mol­ecules are connected by C—H⋯O inter­actions, generating *R*_2_^2^(12) dimers. These dimers are further connected by C—H⋯π inter­actions, linking the mol­ecules into chains running along the *a-*axis direction.

## Related literature   

For background to acridines, see: Kumar *et al.* (2012 ▶). For the biological activity of acridine derivatives, see: Pigatto *et al.* (2011 ▶); Das *et al.* (2011 ▶); Kumar *et al.* (2012 ▶); Prommier *et al.* (2006 ▶) Denny *et al.* (1982 ▶); Baguley & Ferguson (1998 ▶). For the synthesis of acridines, see: Tomar *et al.* (2010 ▶). For related structures, see: Buckleton & Waters (1984 ▶); Chantrapromma *et al.* (2010 ▶). For hydrogen-bond motifs, see: Bernstein *et al.* (1995 ▶).
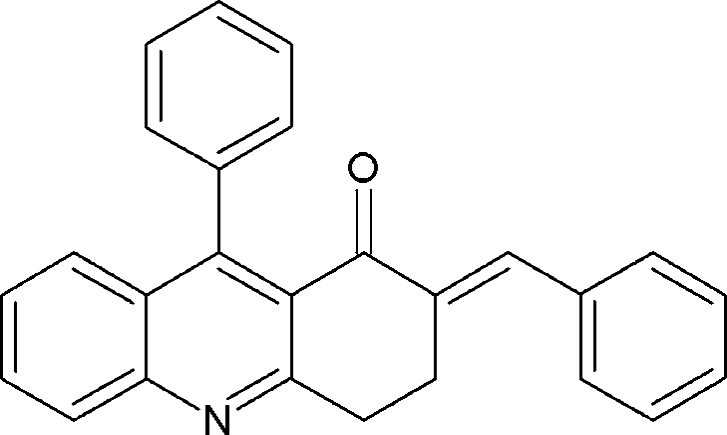


## Experimental   

### 

#### Crystal data  

C_26_H_19_NO
*M* *_r_* = 361.42
Monoclinic, 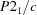

*a* = 9.2222 (3) Å
*b* = 10.7555 (4) Å
*c* = 19.4962 (5) Å
β = 95.503 (2)°
*V* = 1924.90 (11) Å^3^
*Z* = 4
Mo *K*α radiation
μ = 0.08 mm^−1^
*T* = 293 K
0.20 × 0.20 × 0.20 mm

#### Data collection  

Bruker SMART APEXII CCD diffractometer
Absorption correction: multi-scan (*SADABS*; Bruker, 2008 ▶) *T*_min_ = 0.662, *T*_max_ = 0.746
18382 measured reflections
4776 independent reflections
3205 reflections with *I* > 2σ(*I*)
*R*_int_ = 0.029

#### Refinement  

*R*[*F* ^2^ > 2σ(*F* ^2^)] = 0.044
*wR*(*F* ^2^) = 0.131
*S* = 1.00
4776 reflections
254 parameters
H-atom parameters constrained
Δρ_max_ = 0.23 e Å^−3^
Δρ_min_ = −0.16 e Å^−3^

### 

Data collection: *APEX2* (Bruker, 2008 ▶); cell refinement: *SAINT* (Bruker, 2008 ▶); data reduction: *SAINT*; program(s) used to solve structure: *SHELXS97* (Sheldrick, 2008 ▶); program(s) used to refine structure: *SHELXL97* (Sheldrick, 2008 ▶); molecular graphics: *ORTEP-3 for Windows* (Farrugia, 2012 ▶); software used to prepare material for publication: *SHELXL97* and *PLATON* (Spek, 2009 ▶).

## Supplementary Material

Crystal structure: contains datablock(s) I. DOI: 10.1107/S1600536814015943/bt6954sup1.cif

Structure factors: contains datablock(s) I. DOI: 10.1107/S1600536814015943/bt6954Isup2.hkl

Supporting information file. DOI: 10.1107/S1600536814015943/bt6954Isup3.cml

CCDC reference: 1012814

Additional supporting information:  crystallographic information; 3D view; checkCIF report

## Figures and Tables

**Table 1:** Hydrogen-bond geometry (Å, °)

*D*—H⋯*A*	*D*—H	H⋯*A*	*D*⋯*A*	*D*—H⋯*A*
C10—H10*B*⋯O1^i^	0.97	2.58	3.2700 (18)	128
C26—H26⋯*Cg*1^ii^	0.93	2.71	3.577 (18)	156

Symmetry codes: (i) 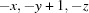; (ii) 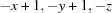.

**Fig. 1.**
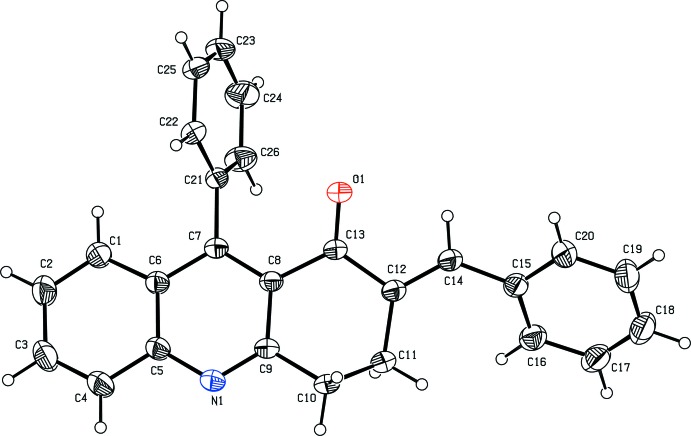
The molecular structure of the title compound with the atom numbering scheme. Displacement ellipsoids are drawn at the 30% probability level. H atoms are presented as small spheres of arbitrary radius.

**Fig. 2.**
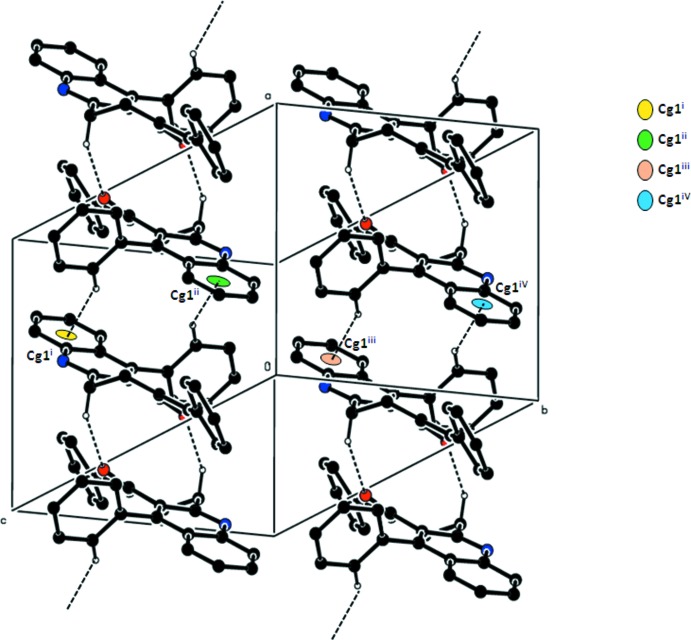
The cystal packing of the title compound showing the intermolecular C—H···O and C—H···π interactions chain running along aaxis, where Cg1 is the centroid of ring atoms C1—C6. Symmetry codes: (i) X,1/2-Y,1/2+Z; (ii) 1-X,-1/2+Y,1/2-Z; (iii) X,3/2-Y,1/2+Z and (iv) 1-X,1/2+Y,1/2-Z.

## supplementary crystallographic information

### S1. Comment

Acridine is structurally related to anthracene with one of the central CH group replaced by nitrogen. Amsacrine which is an acridine derivative is clinically used for the treatment of cancer (Denny *et al.*, 1982; Baguley & Ferguson, 1998). The strong activity of acridine derivatives is due to their ability to intercalate into DNA base pairs and leading to cell cycle arrest and apoptosis (Prommier *et al.*, 2006).

The phenyl (C21—C26) and benzyl (C14—C20) rings deviate from the plane of the acridine system by 72.48 (6) ° and 49.24 (6) °, respectively. The crystal packing is stabilized by intermolecular C—H···O (C10—H10B···O1) interactions generating a *R*^2^_2_(12) ring motif (Bernstein *et al.*, 1995). These dimers are further connected by C—H···π (C26—H26···*Cg*1) interactions generating chains running along the *a*-axis.

### S2. Experimental

A 1:2 molar mixture of 9-phenyl-3,4-dihydroacridin-1(2*H*)-one was treated with aromatic aldehydes in the presence of NaOH and allowed to stir at room temperature for 5–7 h. After completion of the reaction as inferred by the TLC, the mixture was poured into 200 g of crushed ice and neutralized with dil HCl. The precipitate thus formed after adding into crushed ice was filtered off and the residue subjected to column chromatography using petroleum ether: ethyl acetate mixture (3:1) *v*/*v* as eluent and compound obtained as a pale yellow solid.

### S3. Refinement

All H atoms were located in a difference map. Nevertheless, they were positioned geometrically (C—H = 0.93–0.98 Å) and allowed to ride on their parent atoms, with *U*_iso_(H) =1.2*U*_eq_(C).

### Crystal data

**Table:**

C_26_H_19_NO	*F*(000) = 760
*M**_r_* = 361.42	*D*_x_ = 1.247 Mg m^−^^3^*D*_m_ = 1.25 Mg m^−^^3^*D*_m_ measured by not measured
Monoclinic, *P*2_1_/*c*	Mo *K*α radiation, λ = 0.71073 Å
Hall symbol: -P 2ybc	Cell parameters from 4776 reflections
*a* = 9.2222 (3) Å	θ = 2.1–28.3°
*b* = 10.7555 (4) Å	µ = 0.08 mm^−^^1^
*c* = 19.4962 (5) Å	*T* = 293 K
β = 95.503 (2)°	Block, white
*V* = 1924.90 (11) Å^3^	0.20 × 0.20 × 0.20 mm
*Z* = 4	

### Data collection

**Table:**

Bruker SMART APEXII CCD diffractometer	4776 independent reflections
Radiation source: fine-focus sealed tube	3205 reflections with *I* > 2σ(*I*)
Graphite monochromator	*R*_int_ = 0.029
ω and φ scans	θ_max_ = 28.3°, θ_min_ = 2.1°
Absorption correction: multi-scan (*SADABS*; Bruker, 2008)	*h* = −12→10
*T*_min_ = 0.662, *T*_max_ = 0.746	*k* = −13→14
18382 measured reflections	*l* = −25→25

### Refinement

**Table:**

Refinement on *F*^2^	Secondary atom site location: difference Fourier map
Least-squares matrix: full	Hydrogen site location: inferred from neighbouring sites
*R*[*F*^2^ > 2σ(*F*^2^)] = 0.044	H-atom parameters constrained
*wR*(*F*^2^) = 0.131	*w* = 1/[σ^2^(*F*_o_^2^) + (0.0587*P*)^2^ + 0.3326*P*] where *P* = (*F*_o_^2^ + 2*F*_c_^2^)/3
*S* = 1.00	(Δ/σ)_max_ = 0.013
4776 reflections	Δρ_max_ = 0.23 e Å^−^^3^
254 parameters	Δρ_min_ = −0.16 e Å^−^^3^
0 restraints	Extinction correction: *SHELXL97* (Sheldrick, 2008), Fc^*^=kFc[1+0.001xFc^2^λ^3^/sin(2θ)]^-1/4^
Primary atom site location: structure-invariant direct methods	Extinction coefficient: 0.0041 (8)

### Special details

**Table:**

Geometry. All e.s.d.'s (except the e.s.d. in the dihedral angle between two l.s. planes) are estimated using the full covariance matrix. The cell e.s.d.'s are taken into account individually in the estimation of e.s.d.'s in distances, angles and torsion angles; correlations between e.s.d.'s in cell parameters are only used when they are defined by crystal symmetry. An approximate (isotropic) treatment of cell e.s.d.'s is used for estimating e.s.d.'s involving l.s. planes.
Refinement. Refinement of *F*^2^ against ALL reflections. The weighted *R*-factor *wR* and goodness of fit *S* are based on *F*^2^, conventional *R*-factors *R* are based on *F*, with *F* set to zero for negative *F*^2^. The threshold expression of *F*^2^ > σ(*F*^2^) is used only for calculating *R*-factors(gt) *etc*. and is not relevant to the choice of reflections for refinement. *R*-factors based on *F*^2^ are statistically about twice as large as those based on *F*, and *R*- factors based on ALL data will be even larger.

### Fractional atomic coordinates and isotropic or equivalent isotropic displacement parameters (Å^2^)

**Table:**

	*x*	*y*	*z*	*U*_iso_*/*U*_eq_	
C1	0.47972 (17)	0.49247 (14)	0.17309 (8)	0.0542 (4)	
H1	0.4841	0.4062	0.1757	0.065*	
C2	0.5587 (2)	0.56214 (17)	0.22142 (9)	0.0711 (5)	
H2	0.6162	0.5233	0.2569	0.085*	
C3	0.5539 (2)	0.69204 (17)	0.21798 (10)	0.0811 (6)	
H3	0.6089	0.7388	0.2511	0.097*	
C4	0.4701 (2)	0.75058 (15)	0.16689 (9)	0.0677 (5)	
H4	0.4674	0.8370	0.1655	0.081*	
C5	0.38702 (16)	0.68116 (12)	0.11582 (7)	0.0457 (3)	
C6	0.39092 (15)	0.54970 (12)	0.11883 (7)	0.0416 (3)	
C7	0.30846 (14)	0.48187 (11)	0.06585 (6)	0.0376 (3)	
C8	0.23412 (14)	0.54820 (11)	0.01290 (6)	0.0381 (3)	
C9	0.23927 (14)	0.68112 (11)	0.01406 (7)	0.0399 (3)	
C10	0.15959 (17)	0.75179 (12)	−0.04403 (7)	0.0484 (3)	
H10A	0.1996	0.8350	−0.0456	0.058*	
H10B	0.0577	0.7590	−0.0361	0.058*	
C11	0.17193 (18)	0.68716 (12)	−0.11266 (7)	0.0502 (4)	
H11A	0.1159	0.7328	−0.1490	0.060*	
H11B	0.2730	0.6870	−0.1227	0.060*	
C12	0.11714 (15)	0.55543 (12)	−0.11151 (7)	0.0434 (3)	
C13	0.14319 (15)	0.48612 (11)	−0.04483 (6)	0.0399 (3)	
C14	0.04150 (17)	0.49483 (12)	−0.16301 (7)	0.0475 (3)	
H14	0.0134	0.4146	−0.1523	0.057*	
C15	−0.00427 (18)	0.53472 (12)	−0.23384 (7)	0.0488 (3)	
C16	0.0762 (2)	0.61459 (14)	−0.27197 (8)	0.0565 (4)	
H16	0.1647	0.6460	−0.2525	0.068*	
C17	0.0255 (2)	0.64745 (16)	−0.33848 (8)	0.0688 (5)	
H17	0.0809	0.6998	−0.3635	0.083*	
C18	−0.1053 (3)	0.60374 (18)	−0.36787 (9)	0.0773 (6)	
H18	−0.1398	0.6280	−0.4122	0.093*	
C19	−0.1857 (2)	0.52368 (18)	−0.33150 (9)	0.0739 (5)	
H19	−0.2745	0.4935	−0.3515	0.089*	
C20	−0.1349 (2)	0.48783 (15)	−0.26524 (8)	0.0601 (4)	
H20	−0.1886	0.4319	−0.2415	0.072*	
C21	0.31014 (14)	0.34287 (11)	0.06809 (6)	0.0398 (3)	
C22	0.23121 (16)	0.27801 (12)	0.11307 (7)	0.0486 (3)	
H22	0.1776	0.3208	0.1435	0.058*	
C23	0.23196 (19)	0.14904 (14)	0.11288 (9)	0.0607 (4)	
H23	0.1770	0.1056	0.1425	0.073*	
C24	0.3134 (2)	0.08513 (14)	0.06919 (10)	0.0710 (5)	
H24	0.3138	−0.0013	0.0691	0.085*	
C25	0.3942 (2)	0.14968 (16)	0.02578 (10)	0.0766 (5)	
H25	0.4509	0.1068	−0.0033	0.092*	
C26	0.39204 (19)	0.27804 (14)	0.02481 (9)	0.0604 (4)	
H26	0.4463	0.3209	−0.0053	0.072*	
N1	0.31024 (13)	0.74545 (10)	0.06376 (6)	0.0459 (3)	
O1	0.09155 (12)	0.38323 (8)	−0.03732 (5)	0.0517 (3)	

### Atomic displacement parameters (Å^2^)

**Table:**

	*U*^11^	*U*^22^	*U*^33^	*U*^12^	*U*^13^	*U*^23^
C1	0.0594 (9)	0.0455 (8)	0.0552 (8)	0.0054 (7)	−0.0065 (7)	−0.0014 (6)
C2	0.0810 (12)	0.0635 (10)	0.0627 (10)	0.0080 (9)	−0.0252 (9)	−0.0038 (8)
C3	0.1013 (15)	0.0616 (11)	0.0722 (11)	0.0025 (10)	−0.0351 (11)	−0.0180 (9)
C4	0.0883 (13)	0.0435 (8)	0.0671 (10)	0.0003 (8)	−0.0153 (9)	−0.0135 (7)
C5	0.0519 (8)	0.0371 (7)	0.0476 (7)	0.0009 (6)	0.0017 (6)	−0.0063 (6)
C6	0.0442 (7)	0.0374 (7)	0.0431 (7)	0.0019 (6)	0.0029 (6)	−0.0019 (5)
C7	0.0401 (7)	0.0310 (6)	0.0421 (6)	0.0006 (5)	0.0059 (5)	0.0004 (5)
C8	0.0424 (7)	0.0301 (6)	0.0419 (7)	−0.0008 (5)	0.0045 (5)	0.0012 (5)
C9	0.0441 (7)	0.0298 (6)	0.0460 (7)	−0.0005 (5)	0.0054 (6)	0.0008 (5)
C10	0.0619 (9)	0.0281 (6)	0.0537 (8)	−0.0006 (6)	−0.0023 (7)	0.0052 (6)
C11	0.0655 (9)	0.0372 (7)	0.0470 (7)	−0.0054 (6)	0.0013 (7)	0.0077 (6)
C12	0.0527 (8)	0.0340 (6)	0.0434 (7)	0.0007 (6)	0.0039 (6)	0.0030 (5)
C13	0.0467 (7)	0.0299 (6)	0.0428 (7)	−0.0002 (5)	0.0028 (6)	0.0022 (5)
C14	0.0638 (9)	0.0346 (7)	0.0437 (7)	0.0006 (6)	0.0037 (6)	0.0023 (5)
C15	0.0669 (9)	0.0376 (7)	0.0415 (7)	0.0072 (7)	0.0042 (7)	−0.0024 (6)
C16	0.0744 (11)	0.0466 (8)	0.0493 (8)	0.0060 (7)	0.0093 (7)	0.0015 (7)
C17	0.1057 (15)	0.0537 (9)	0.0488 (9)	0.0118 (10)	0.0160 (10)	0.0080 (7)
C18	0.1155 (17)	0.0686 (12)	0.0457 (9)	0.0241 (11)	−0.0039 (10)	0.0050 (8)
C19	0.0860 (13)	0.0770 (12)	0.0544 (10)	0.0099 (10)	−0.0154 (9)	−0.0101 (9)
C20	0.0771 (11)	0.0542 (9)	0.0477 (8)	−0.0002 (8)	0.0000 (8)	−0.0060 (7)
C21	0.0437 (7)	0.0315 (6)	0.0429 (7)	0.0038 (5)	−0.0031 (6)	0.0013 (5)
C22	0.0569 (9)	0.0395 (7)	0.0485 (8)	0.0022 (6)	0.0000 (6)	0.0072 (6)
C23	0.0715 (11)	0.0423 (8)	0.0648 (10)	−0.0094 (7)	−0.0124 (8)	0.0168 (7)
C24	0.0966 (14)	0.0291 (7)	0.0817 (12)	0.0063 (8)	−0.0204 (11)	0.0012 (8)
C25	0.1012 (15)	0.0443 (9)	0.0846 (13)	0.0234 (9)	0.0111 (11)	−0.0074 (9)
C26	0.0708 (10)	0.0422 (8)	0.0705 (10)	0.0103 (7)	0.0191 (8)	0.0008 (7)
N1	0.0549 (7)	0.0322 (5)	0.0499 (6)	0.0004 (5)	0.0015 (5)	−0.0035 (5)
O1	0.0673 (7)	0.0343 (5)	0.0514 (6)	−0.0106 (4)	−0.0052 (5)	0.0058 (4)

### Geometric parameters (Å, º)

**Table:**

C1—C2	1.359 (2)	C13—O1	1.2191 (15)
C1—C6	1.4151 (19)	C14—C15	1.4689 (18)
C1—H1	0.9300	C14—H14	0.9300
C2—C3	1.399 (2)	C15—C20	1.393 (2)
C2—H2	0.9300	C15—C16	1.395 (2)
C3—C4	1.356 (2)	C16—C17	1.381 (2)
C3—H3	0.9300	C16—H16	0.9300
C4—C5	1.411 (2)	C17—C18	1.369 (3)
C4—H4	0.9300	C17—H17	0.9300
C5—N1	1.3682 (17)	C18—C19	1.377 (3)
C5—C6	1.4154 (18)	C18—H18	0.9300
C6—C7	1.4236 (17)	C19—C20	1.386 (2)
C7—C8	1.3818 (17)	C19—H19	0.9300
C7—C21	1.4957 (17)	C20—H20	0.9300
C8—C9	1.4304 (17)	C21—C26	1.3750 (19)
C8—C13	1.4949 (17)	C21—C22	1.3813 (19)
C9—N1	1.3132 (16)	C22—C23	1.387 (2)
C9—C10	1.4970 (18)	C22—H22	0.9300
C10—C11	1.522 (2)	C23—C24	1.373 (3)
C10—H10A	0.9700	C23—H23	0.9300
C10—H10B	0.9700	C24—C25	1.369 (3)
C11—C12	1.5051 (18)	C24—H24	0.9300
C11—H11A	0.9700	C25—C26	1.381 (2)
C11—H11B	0.9700	C25—H25	0.9300
C12—C14	1.3361 (18)	C26—H26	0.9300
C12—C13	1.4978 (17)		
			
C2—C1—C6	120.76 (14)	O1—C13—C12	121.60 (12)
C2—C1—H1	119.6	C8—C13—C12	117.57 (11)
C6—C1—H1	119.6	C12—C14—C15	130.26 (13)
C1—C2—C3	120.33 (15)	C12—C14—H14	114.9
C1—C2—H2	119.8	C15—C14—H14	114.9
C3—C2—H2	119.8	C20—C15—C16	118.07 (14)
C4—C3—C2	120.80 (15)	C20—C15—C14	117.78 (14)
C4—C3—H3	119.6	C16—C15—C14	124.14 (14)
C2—C3—H3	119.6	C17—C16—C15	120.55 (17)
C3—C4—C5	120.37 (15)	C17—C16—H16	119.7
C3—C4—H4	119.8	C15—C16—H16	119.7
C5—C4—H4	119.8	C18—C17—C16	120.70 (18)
N1—C5—C4	117.62 (13)	C18—C17—H17	119.7
N1—C5—C6	123.01 (12)	C16—C17—H17	119.7
C4—C5—C6	119.32 (13)	C17—C18—C19	119.74 (16)
C1—C6—C5	118.43 (12)	C17—C18—H18	120.1
C1—C6—C7	123.37 (12)	C19—C18—H18	120.1
C5—C6—C7	118.18 (12)	C18—C19—C20	120.23 (18)
C8—C7—C6	118.02 (11)	C18—C19—H19	119.9
C8—C7—C21	122.74 (11)	C20—C19—H19	119.9
C6—C7—C21	119.20 (11)	C19—C20—C15	120.66 (17)
C7—C8—C9	119.39 (11)	C19—C20—H20	119.7
C7—C8—C13	122.29 (11)	C15—C20—H20	119.7
C9—C8—C13	118.27 (11)	C26—C21—C22	119.17 (13)
N1—C9—C8	123.48 (12)	C26—C21—C7	119.59 (12)
N1—C9—C10	117.68 (11)	C22—C21—C7	121.24 (12)
C8—C9—C10	118.84 (11)	C21—C22—C23	120.02 (14)
C9—C10—C11	111.14 (11)	C21—C22—H22	120.0
C9—C10—H10A	109.4	C23—C22—H22	120.0
C11—C10—H10A	109.4	C24—C23—C22	120.35 (16)
C9—C10—H10B	109.4	C24—C23—H23	119.8
C11—C10—H10B	109.4	C22—C23—H23	119.8
H10A—C10—H10B	108.0	C25—C24—C23	119.48 (15)
C12—C11—C10	111.30 (11)	C25—C24—H24	120.3
C12—C11—H11A	109.4	C23—C24—H24	120.3
C10—C11—H11A	109.4	C24—C25—C26	120.52 (17)
C12—C11—H11B	109.4	C24—C25—H25	119.7
C10—C11—H11B	109.4	C26—C25—H25	119.7
H11A—C11—H11B	108.0	C21—C26—C25	120.43 (16)
C14—C12—C13	115.98 (12)	C21—C26—H26	119.8
C14—C12—C11	126.86 (12)	C25—C26—H26	119.8
C13—C12—C11	117.09 (11)	C9—N1—C5	117.84 (11)
O1—C13—C8	120.83 (11)		
			
C6—C1—C2—C3	−0.3 (3)	C14—C12—C13—O1	−3.7 (2)
C1—C2—C3—C4	0.4 (3)	C11—C12—C13—O1	173.38 (13)
C2—C3—C4—C5	−0.5 (3)	C14—C12—C13—C8	176.83 (12)
C3—C4—C5—N1	−176.88 (17)	C11—C12—C13—C8	−6.04 (18)
C3—C4—C5—C6	0.6 (3)	C13—C12—C14—C15	179.30 (14)
C2—C1—C6—C5	0.4 (2)	C11—C12—C14—C15	2.5 (3)
C2—C1—C6—C7	178.48 (15)	C12—C14—C15—C20	−148.09 (16)
N1—C5—C6—C1	176.83 (13)	C12—C14—C15—C16	33.2 (2)
C4—C5—C6—C1	−0.5 (2)	C20—C15—C16—C17	1.1 (2)
N1—C5—C6—C7	−1.4 (2)	C14—C15—C16—C17	179.87 (14)
C4—C5—C6—C7	−178.70 (14)	C15—C16—C17—C18	0.9 (3)
C1—C6—C7—C8	−175.42 (13)	C16—C17—C18—C19	−1.6 (3)
C5—C6—C7—C8	2.67 (18)	C17—C18—C19—C20	0.3 (3)
C1—C6—C7—C21	2.24 (19)	C18—C19—C20—C15	1.8 (3)
C5—C6—C7—C21	−179.67 (12)	C16—C15—C20—C19	−2.5 (2)
C6—C7—C8—C9	−1.66 (18)	C14—C15—C20—C19	178.72 (14)
C21—C7—C8—C9	−179.24 (12)	C8—C7—C21—C26	74.02 (18)
C6—C7—C8—C13	−179.36 (11)	C6—C7—C21—C26	−103.53 (15)
C21—C7—C8—C13	3.06 (19)	C8—C7—C21—C22	−106.21 (15)
C7—C8—C9—N1	−0.88 (19)	C6—C7—C21—C22	76.24 (16)
C13—C8—C9—N1	176.92 (12)	C26—C21—C22—C23	−1.8 (2)
C7—C8—C9—C10	179.33 (12)	C7—C21—C22—C23	178.44 (13)
C13—C8—C9—C10	−2.87 (18)	C21—C22—C23—C24	1.4 (2)
N1—C9—C10—C11	141.67 (13)	C22—C23—C24—C25	0.0 (3)
C8—C9—C10—C11	−38.53 (17)	C23—C24—C25—C26	−1.2 (3)
C9—C10—C11—C12	56.53 (17)	C22—C21—C26—C25	0.7 (2)
C10—C11—C12—C14	142.07 (15)	C7—C21—C26—C25	−179.55 (15)
C10—C11—C12—C13	−34.70 (18)	C24—C25—C26—C21	0.8 (3)
C7—C8—C13—O1	24.48 (19)	C8—C9—N1—C5	2.25 (19)
C9—C8—C13—O1	−153.24 (13)	C10—C9—N1—C5	−177.96 (12)
C7—C8—C13—C12	−156.08 (12)	C4—C5—N1—C9	176.28 (13)
C9—C8—C13—C12	26.19 (17)	C6—C5—N1—C9	−1.1 (2)

### Hydrogen-bond geometry (Å, º)

**Table:**

*D*—H···*A*	*D*—H	H···*A*	*D*···*A*	*D*—H···*A*
C10—H10*B*···O1^i^	0.97	2.58	3.2700 (18)	128
C26—H26···*Cg*1^ii^	0.93	2.71	3.577 (18)	156

Symmetry codes: (i) −*x*, −*y*+1, −*z*; (ii) −*x*+1, −*y*+1, −*z*.
